# Intervertebral disc degeneration induced by long-segment in-situ immobilization: a macro, micro, and nanoscale analysis

**DOI:** 10.1186/s12891-018-2235-z

**Published:** 2018-08-28

**Authors:** Yan-Jun Che, Hai-Tao Li, Ting Liang, Xi Chen, Jiang-Bo Guo, Hua-Ye Jiang, Zong-Ping Luo, Hui-Lin Yang

**Affiliations:** 1 Orthopaedic Institute, Department of Orthopaedics, The First Affiliated Hospital of SooChow University, 708 Renmin Rd, Suzhou, Jiangsu 215006 People’s Republic of China; 2 Department of Orthopedics, Peace Hospital Affiliated to Changzhi Medical College, Changzhi, Shanxi People’s Republic of China

**Keywords:** Intervertebral disc degeneration, Immobilization, Cervical spine, Fixation, Biomechanics, Rat model

## Abstract

**Background:**

Cervical spine fixation or immobilization has become a routine treatment for spinal fracture, dislocation, subluxation injuries, or spondylosis. The effects of immobilization of intervertebral discs of the cervical spine is unclear. The goal of this study was to evaluate the effects of long-segment in-situ immobilization of intervertebral discs of the caudal vertebra, thereby simulating human cervical spine immobilization.

**Methods:**

Thirty-five fully grown, male Sprague-Dawley rats were used. Rats were randomly assigned to one of five groups: Group A, which served as controls, and Groups B, C, D, and E, in which the caudal vertebrae were in-situ immobilized using a custom-made external device that fixed four caudal vertebrae (Co7-Co10). After 2 weeks, 4 weeks, 6 weeks, and 8 weeks of in-situ immobilization, the caudal vertebrae were harvested, and the disc height, the T2 signal intensity of the discs, disc morphology, the gene expression of discs, and the structure and the elastic modulus of discs was measured.

**Results:**

The intervertebral disc height progressively decreased, starting at the 6th week. At week 6 and week 8, disc degeneration was classified as grade III, according to the modified Pfirrmann grading system criteria. Long-segment immobilization altered the gene expression of discs. The nucleus pulposus showed a typical cell cluster phenomenon over time. The annulus fibrosus inner layer began to appear disordered with fissure formation. The elastic modulus of collagen fibrils within the nucleus pulposus was significantly decreased in rats in group E compared to rats in group A (*p* < 0.05). On the contrary, the elastic modulus within the annulus was significantly increased in rats in group E compared to rats in group A (*p* < 0.05).

**Conclusion:**

Long-segment in-situ immobilization caused target disc degeneration, and positively correlated with fixation time. The degeneration was not only associated with changes at the macroscale and microscale, but also indicated changes in collagen fibrils at the nanoscale. Long-segment immobilization of the spine (cervical spine) does not seem to be an innocuous strategy for the treatment of spine-related diseases and may be a predisposing factor in the development of the symptomatic spine.

## Background

Previous studies have shown that disc degeneration is closely associated with low back pain (LBP) [1, 2]. The mechanical environment of the intervertebral disc (IVD) at least in part determines the rate of disc degeneration. However, in terms of overload and “wear and tear” theory, the mechanical environment around the nucleus pulposus (NP) and annulus fibrosus (AF) is more harsher, so that damage to the disc cannot be completely recovered [3]. In previous studies, the degeneration model of articular cartilage induced by immobilization has been clearly demonstrated [4–9], however, the IVD degeneration induced by immobilization in human spine is still controversial. Due to fractures of the cervical spine, dislocations and cervical spondylosis, in order to restore the stability of the spine (cervical spine), a cervical extension collar, brace, or Halo-vest immobilization may be required. However, after release of immobilization, complications may remain, including stiffness of the neck and restricted movement [10, 11]. It cannot be ruled out that the immobilization apparatus was a causal factor to the complications, and the underlying mechanism that is a factor in these complications has yet to be identified. In this study, three caudal vertebrae (including two discs) were fixed, this method manufactured reconstitution alterations that were similar to those found using static compression, but with less changes in configuration and synthesis [12]. Immobilization using fixation of two caudal vertebrae, including one disc, downregulated the expression of anabolic genes [13]. The hypothesis that movement increases the conveyance of nutrients and metabolites in the disc was recently investigated, and it was found that essential nutrients are transported through diffusion and convection [14]. Moreover, it was suggested that a “pumping” effect would accelerate the conveyance of molecules larger than that of sulfate ions. Several studies have shown the effect induced by short-segment immobilization (usually fixation is less than/or equal to three caudal vertebrae, including two discs). Because of the anatomical features of the cervical vertebrae (C4–7 intervertebral discs is an easier segment to degenerate or protrude), cervical spine immobilization or fixation (a brace or Halo-Vest fixator) is more inclined to overall fixation (similar to long tube fixation). However, the effect of long-segment in-situ immobilization and the underlying mechanism involved in the onset of complications remains unknown.

The purpose of this study is to identify the effect of long-segment in-situ immobilization (fixation of four caudal vertebrae, including three discs) caused by biochemical composition, gene expression, matrix reconstruction, and cellular responses, and to assess the effects of long-segment in-situ immobilization on intervertebral discs of the caudal vertebra, thereby simulating human cervical spine immobilization. Therefore, we hypothesize: 1) intervertebral disc degeneration is induced by long-segment in-situ immobilization; 2) intervertebral disc degeneration positively correlates with immobilization time; 3) the mechanism involved in complications of cervical spine long-segment in-situ immobilization can be explained at least in part by intervertebral disc degeneration.

## Methods

**Fig. 1:**
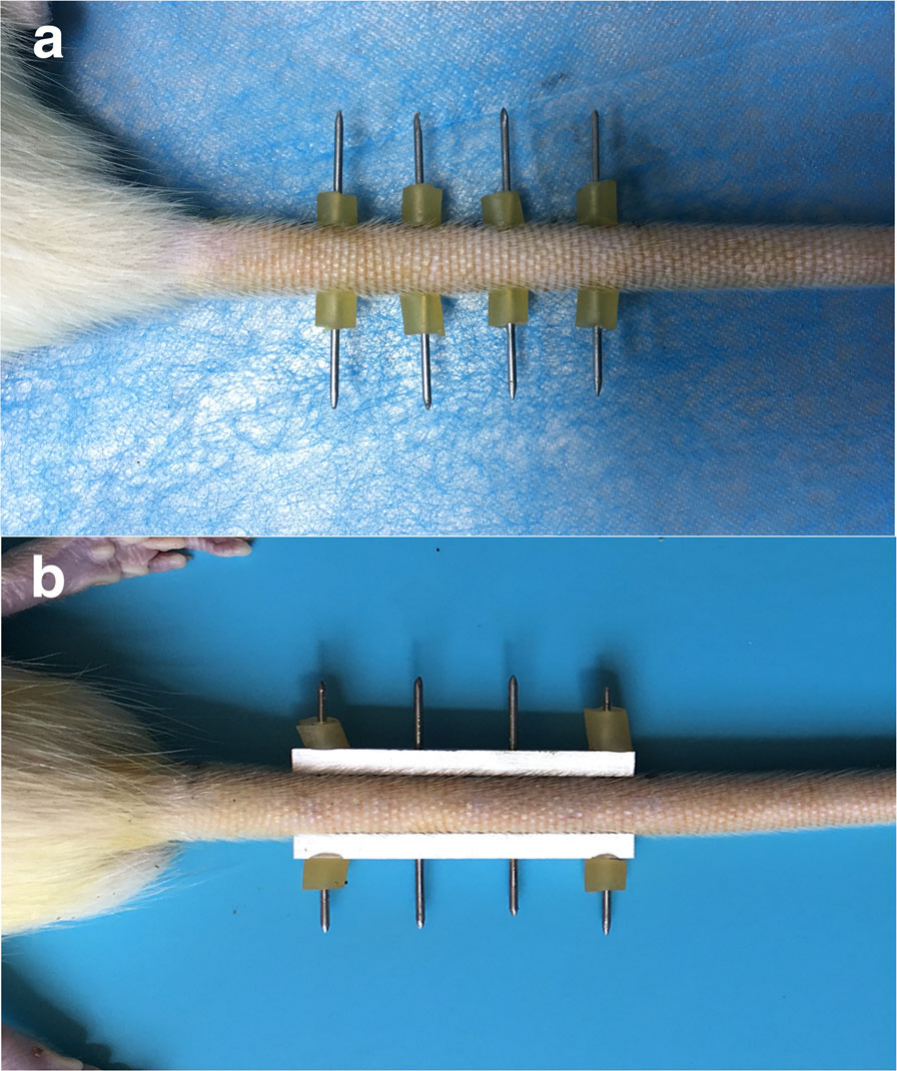
Animal model. **a**. the caudal vertebrae were instrumented with K-wires only, which served as controls. **b**. the caudal vertebrae were immobilized using a custom-made external device to fix four caudal vertebrae (Co7-Co10). Four K-wires (50 mm in length and 1.2 mm in diameter) were fixed in parallel using two aluminum alloy cuboids (43 mm in length, 4 mm in width, net weight 5.0 g, the hole spacing is 12 mm), which do not compress or stretch the experimental discs

In this study, thirty-five fully grown, 3-month-old male Sprague-Dawley rats were used [15]. Animals were randomly assigned to one of five groups (Table 1). Group A (*n* = 7 rats, the caudal vertebrae were instrumented with K-wires only, Fig. 1a) which served as controls. In the other four groups, vertebrae were immobilized using a custom-made external device to fix four caudal vertebrae (Co7-Co10). After 2 weeks (*n* = 7 rats, Group B), 4 weeks (*n* = 7 rats, Group C), 6 weeks (*n* = 7 rats, Group D), and 8 weeks (*n* = 7 rats, Group E) of immobilization, animals were euthanized and the caudal vertebrae were harvested for further analysis. The disc space was measured using radiography [16], and MRI qualitative analysis according to the modified Pffirmann scale [17]. Next, an experienced radiologist and a senior director experienced in spines analyzed the MRI-derived data (Table 2). Histological evaluation was based on the grading system developed by Han et al. [18]. Disc anabolic (collagen I, collagen II, aggrecan) and expression of catabolic genes (MMP3, MMP13, ADAMTs-4) were measured, respectively. Four K-wires were fixed in parallel using two aluminum alloy cuboids, which do not compress or stretch the experimental disc (Fig. 1b).

**Table 1:** Summary of study design

Group	Instrumented level (Co7-Co10)	No. of Animals
A (Control)	Instrumented with K-wires only	7
B	Imm-2 week	7
C	Imm-4 week	7
D	Imm-6 week	7
E	Imm-8 week	7

*Imm* indicates immobilization, *Co* indicates Coccygeal spine

**Table 2:** Modified Magnetic Resonance Imaging Pfirrmann Grading

Grade	Structural Changes Within NP	Signal Intensity	IVDH
I	Homogenous and bright	Hyperintense	Normal
II	Heterogeneous	Intermediate	Normal
III	Heterogeneous and gray	Intermediate	Decreased
IV	Heterogeneous and black	Hypointense	Decreased or collapsed

*NP* indicates nucleus pulposus, *IVDH* indicates Intervertebral Disc Height

### Histological analysis

After immobilization for 2, 4, 6, and 8 weeks, respectively, the rats were examined by X-ray and MRI analysis. Then, the animals were euthanized by an excess of isoflurane (isoflurane, RWD Life science co. Shenzhen, China). The target discs Co7-Co8, Co8-Co9, Co9-Co10 were harvested, fixed in 10% buffered formalin solution (Shanghai Yuanye Bio-Technology Co. Ltd., Shanghai, China) for 24 h, and decalcified in 10% ethylenediaminetetraacetic acid (EDTA) (Biosharp, Hefei, China) for 30 days. The discs were then paraffin-embedded (Leica, Richmond, USA), and sectioned using a histotome (Leica, Heidelberger, Germany). For histological analysis, sections (5 μm) were stained with hematoxylin/eosin (Beijing BiotoppedScience & Technology Co. Ltd., Beijing, China), whereas for AFM scanning, 10-20 μm sections were used. Histological images were visualized using a binocular microscope (XSP-2CA, Shanghai, China), and changes in the AF were assessed using a grading scale of the stained images at a magnification of 200× as described by Masuda et al. [16]. The number of cells in the NP was scored by counting from the hematoxylin/eosin stained images at a magnification of 400 ×.

### Gene expression analysis by RT-PCR

For each specimen, (35 rats, 1 disc levels, AF and NP; *n* = 70), total RNA was extracted using TRIzol® reagent and a total of 1 μg of total RNA was reverse-transcribed using the Revert Aid First Strand cDNA Synthesis Kit (Thermo Fisher Scientific; Waltham, MA, USA). To quantify mRNA expression, an amount of cDNA that was equivalent to 50 ng of total RNA was amplified by real-time PCR using the iTaqTM Universal SYBR® Green Supermix kit (Bio-Rad, Hercules, CA, USA) [19]. Transcript levels of anabolic genes (collagen I, collagen II, aggrecan) and catabolic genes (MMP3, MMP13, ADAMTs-4) were evaluated. GAPDH served as an internal standard. Primer sequences are presented in Table 3. Rt-PCR was performed on a CFX96TM rt-PCR System (Bio-Rad, Hercules, CA, USA) following the manufacturer’s guidelines. Relative transcript levels were calculated as χ = 2-ΔΔCt, in which ΔΔCt = ΔE - ΔC, ΔE = Ctexp - CtGAPDH, and ΔC = Ctct1 - CtGAPDH [19].

**Table 3:** Primers and Probes for Real-Time RT-PCR

Target Gene	Sequence (5′→3′)
GAPDH	
Forward:	AGA CAG CCG CAT CTT CTT GT
Reverse:	TAC TCA GCA CCA GCA TCA CC
Collagen I	
Forward:	ATG TTC AGC TTT GTG GAC
Reverse:	GGA TGC CAT CTT GTC CAG
Collagen II	
Forward:	CCT GGA CCC CGT GGC AGA GA
Reverse:	CAG CCA TCT GGG CTG CAA AG
Aggrecan	
Forward:	AGG ATG GCT TCC ACC AGT GC
Reverse:	TGC GTA AAA GAC CTC ACC CTC C
MMP-3	
Forward:	TCT TCC TCT GAA ACT TGG CG
Reverse:	AGT GCT TCT GAA TGT CCT TCG
MMP-13	
Forward:	GCA GCT CCA AAG GCT ACA A
Reverse:	CAT CAT CTG GGA GCA TGA AA
ADAMTS-4	
Forward:	CTT CGC TGA GTA GAT TCG TGG
Reverse:	AGT TGA CAG GGT TTC GGA TG

### AFM imaging and nano-mechanical testing

AFM scanner (Dimension ICON, Bruker, USA) was used at atmospheric pressure [20]. The structure and the elastic modulus of individual collagen fibrils within intervertebral discs Co9-Co10 were tested at the nanoscale using AFM in week 2, 4, 6, and 8, respectively. A total of thirty-five collagen fibrils from each rat were tested. Both AFM imaging and nano-mechanical testing were conducted at a scanning rate of 1 Hz using a Scan Asyst-Air probe, a curvature radius of 5 nm, and a force constant of 0.4 N/m.

### Statistical analysis

Experimental data are presented as the mean ± standard deviation (SD). Significant differences between study groups were obtained by using a one-way analysis of variance (ANOVA) with Fisher’s Partial Least-Squares Difference (PLSD) to analyze the influence of immobilization loading and time. Statistical significance was set at *p* ≤ 0.05.

## Results

All 35 rats successful completed the 8 week study. At the beginning of the study, the average body weight was 400 g. Body weight increased to 405 g after 28 days, and was 411 g at the end of the study. This indicated that rats gained weight over time and that the surgery did not affect normal growth and development. The device that was used for immobilization weighted roughly 5.0 g and was well tolerated by the rats, as evidenced by their ability to lift and easily move their tails with the devices attached. Although the segments within the apparatus were largely immobilized, rats were able to move and control their tails both proximal and distal to the devices.

### Histology and morphology

**Fig. 2:**
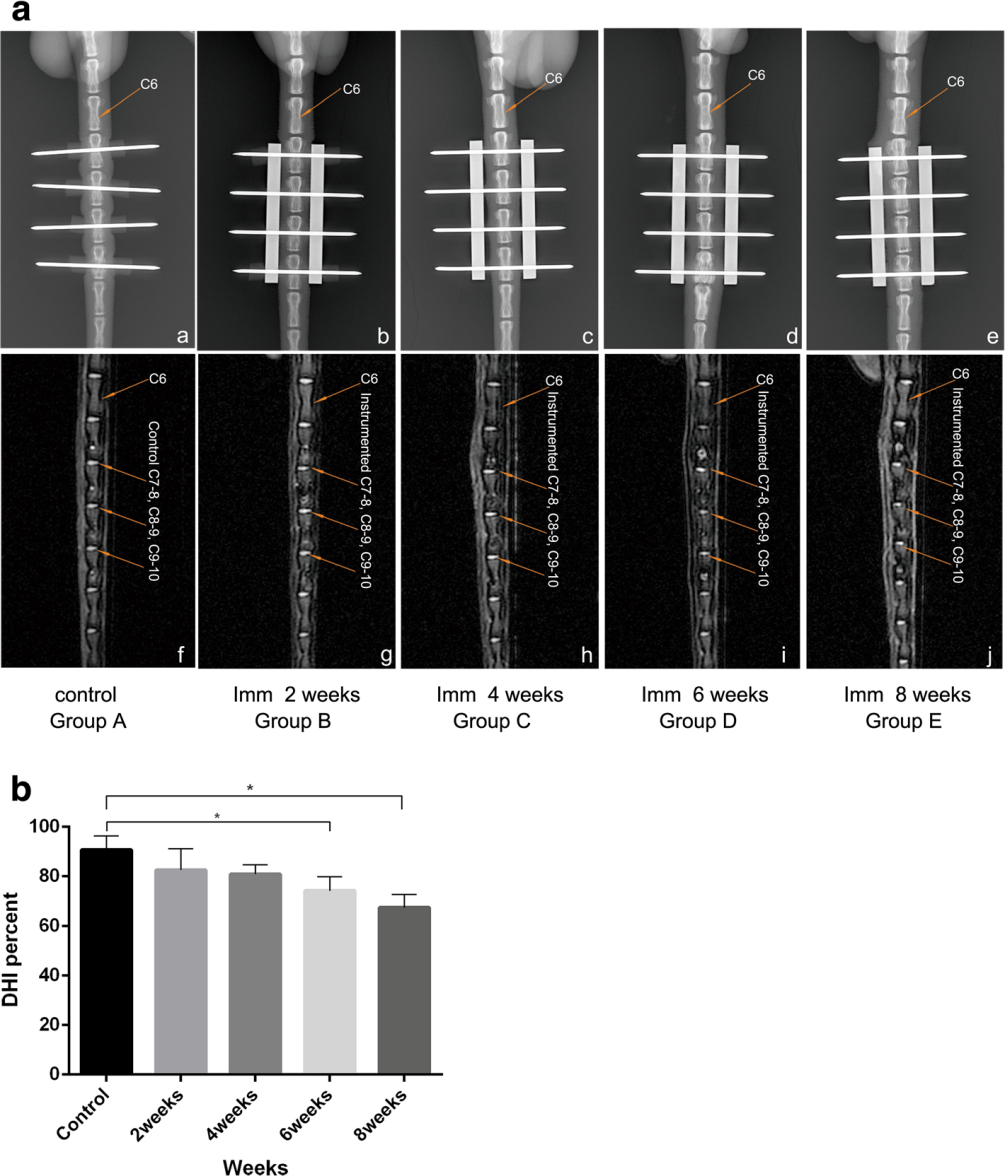
A. The disc space and T2 signal intensity were measured using radiography and MRI Scans. Radiographs: (a~e) were obtained under anesthesia using a digital, self-contained cabinet x-ray machine (exposure time: 10 s, 26 kV). a (Group A) which served as controls, b~e (Group B~E) as shown in the figure, over time, progressive loss of disc height, d and e more obvious. MRI Scans: (f~j) (Scanning Sequence: FRFSE-XL, Slice thickness:1.4 mm, Interlayer Spacing: 5 mm) uses magnetic waves to create pictures to determine nucleus pulposus size and hydration status according to T2 signal intensity. Over time, the T2 signal intensity progressive decrease, as described above, i and j more serious. The IVD degeneration was classified as grade III. Imm indicates immobilization. B. The intervertebral disc height assessment based on radiographs. In control group (group A), imm-2 weeks (group B), and imm-4 weeks (group C), the intervertebral disc space height was slightly decreased, postoperatively, and this reduction was significant starting at the 6th week (group D). (*) indicates significant difference from other groups discs (*p* < 0.05)

The intervertebral disc height progressively decreased with time. A significant decrease was observed in rats in group D and group E with time as shown in Fig. 2A. The percentage of intervertebral disc space height in groups A was significantly higher compared to that in group D and E (Fig. 2B) (*p* < 0.05). The disc thickness was significantly different between rats in group A compared to rats in group D (*p* = 0.021), group A vs group E (*p* < 0.0001), and group B vs group E (*p* = 0.028). At 8 weeks after spine immobilization, modified Pfirrmann grades of I, II, III, and IV were found in 12, 9, 14, and 0 rats, respectively. At week 6 and 8, the intervertebral disc degeneration was classified as grade III, based on the modified Pfirrmann grading system criteria. A total of 14 out of 35 discs had deteriorated between day 0 and 8 weeks after immobilization. A modified Pfirrmann grade of IV was not found in any of the animals examined.

**Fig. 3:**
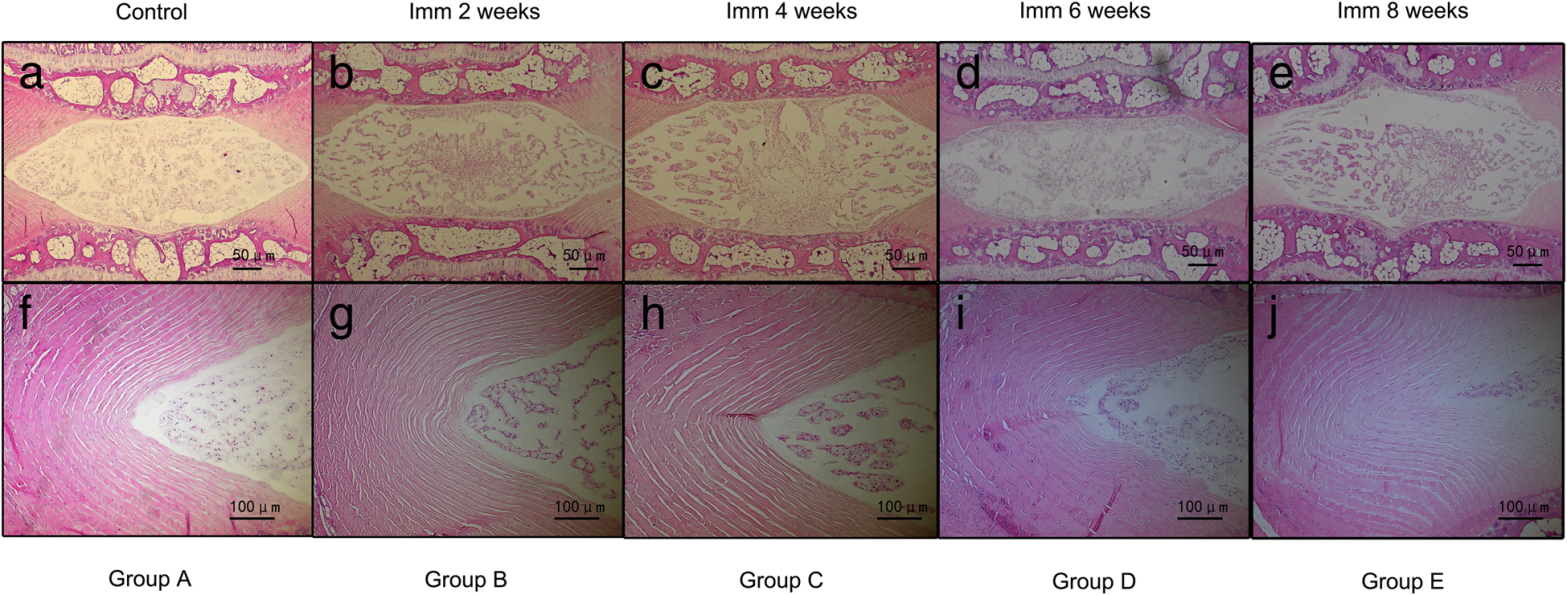
Histological assessments (Hematoxylin/Eosin stain). Histological section demonstrating early degenerative changes of in-situ immobilization intervertebral disc after 2~ 8 weeks. 1. (a~e), Intervertebral disc at low magnification (50×). The most prominent change in this specimen (b~e) is the cluster formation of the nucleus pulposus cells becomes obvious compared to control group A (a). Cells within these clusters kept their typical morphological structure with stellar-shape dnuclei and a vacuolated cytoplasm. 2. Higher magnification of IVD section shown in figure f~j (100×), the inner layer of AF appear to be progressive disorders and hyperplasia (j). Close to the NP border, the AF becomes infiltrated with chondrocyte-resembling cells. AF indicates annulus fibrosus; NP indicates nucleus pulposus; Imm indicates immobilization

**Fig. 4:**
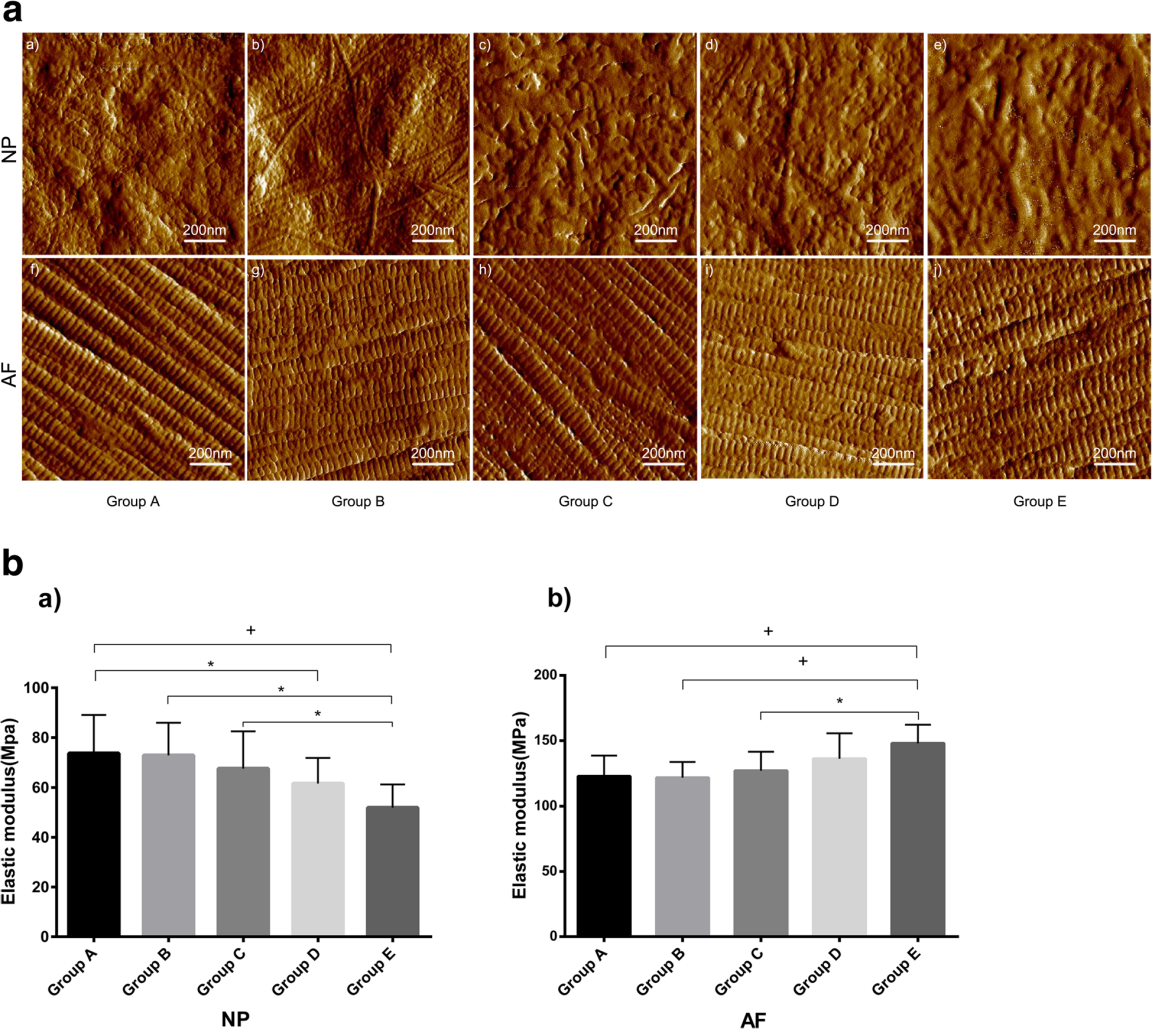
A. AFM was used to observe the microstructure of AF and NP collagen fibers. The representative AFM images of collagen fibrils in AF and NP of the control group and the experimental group after bearing immobilization for 2, 4, 6 and 8 weeks, respectively. The top row represents the AFM image of the NP (a-e), and the image in the lower row represents the AF AFM image (f-j). AFM indicates Atomic force microscopy; AF indicates annulus fibrosus; NP indicates nucleus pulposus. B. The average elastic modulus of collagen fibrils with different immobilization duration. a).The elastic modulus of NP were analyzed. b).The elastic modulus of AF were analyzed. (*) indicates significant difference from other groups discs (*p* ≤ 0.05), (+)indicates significant difference from other groups (*p* ≤ 0.001)

Histological assessments were performed based on the grading system [18]. Previous studies have shown degeneration changes in immobilization IVD [12, 13, 21]. The histological changes found in this study were in line with the changes described by other groups [13, 21]. However, no significant decrease was found in the number of cells in the nucleus pulposus among rats in group A, B, C, D, and E (*p* = 0.370). In contrast, the number of cells in the nucleus pulposus was slightly increased over time in rats in group D and E (*p* > 0.05). Moreover, the cluster formation of the nucleus pulposus cells became obvious. Cells within these clusters maintained their typical morphology with a polymorphonuclear shape, intracellular vacuoles, and stellar nuclei. Moreover, the Extracellular Matrix (ECM) progressively increased and the granules were shriveled. The inner layer of AF appear to be progressive disorders and hyperplasia (Fig. 3f-j). Round or ovoid-shaped chondrocytes infiltrated the AF, leading to the formation of cartilage-resembling tissue bordering the NP. This cartilage-resembling tissue could clearly be distinguished from the NP, displaying a different cell morphology and a matrix that was more intensely stained by hematoxylin/eosin (Fig. 3a-j). Characteristic AFM images of collagen fibrils from IVD of NP and AF for every group are presented in Fig. 4A. Within the NP, the elastic modulus of collagen fibrils progressively decreased (Fig. 4Ba), and was significantly different in rats in group A compared to rats in group E (*p* < 0.001), group A vs group D (*p* < 0.05), group B vs group E (*p* < 0.05), and group C vs group E (*p* < 0.05). Moreover, a gradual increase was found within the AF (Fig. 4Bb), and was significantly different between the elastic modulus of collagen fibrils of rats in group A compared to rats in group E (*p* < 0.001), group B vs group E (*p* < 0.001), and group C vs group E (*p* < 0.05).

### Gene expression

**Fig. 5:**
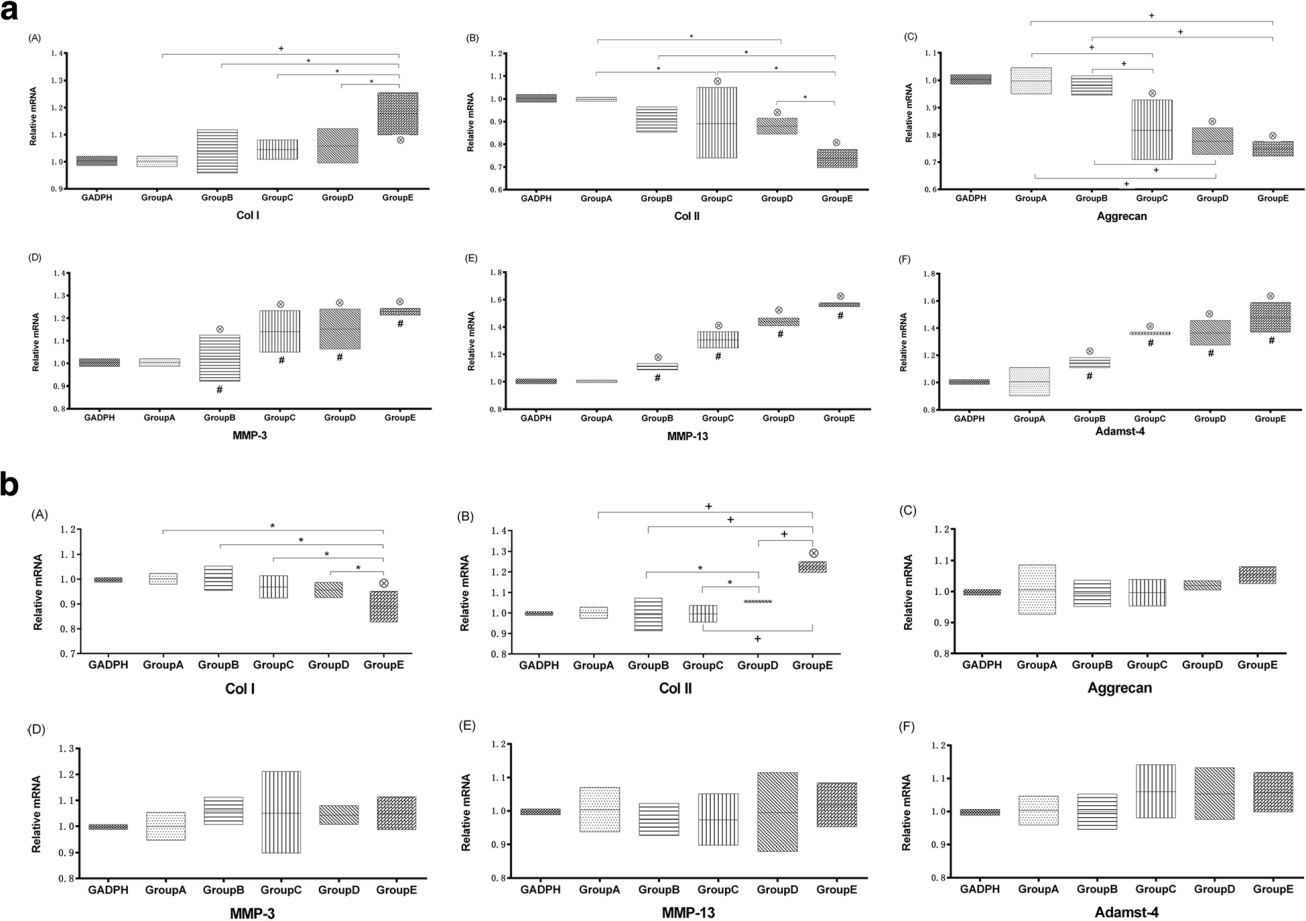
A. The anabolic and catabolic gene expression of NP. mRNA levels of NP in the disc normalized to endogenous control (GAPDH) and internal controls. Relative transcript levels were calculated as χ = 2-ΔΔCt, in which ΔΔCt = ΔE - ΔC, ΔE = Ctexp - CtGAPDH, and ΔC = Ctct1 – CtGAPDH. A~C are shown for anabolic genes in the NP, catabolic genes as shown in the D~F. (⊗) indicates a significant difference from the internal controls (*p* ≤ 0.05) and (#) indicates that from other groups (one-way ANOVA with Fischer PLSD post hoc *p* ≤ 0.05), and (*)indicates a significant difference (*p* ≤ 0.05), and (+) indicates a significant difference (*p* ≤ 0.001). B. The anabolic and catabolic gene expression of AF. mRNA levels of AF in the disc normalized to endogenous control (GAPDH) and internal controls. Relative transcript levels were calculated as χ = 2-ΔΔCt, in which ΔΔCt = ΔE - ΔC, ΔE = Ctexp - CtGAPDH, and ΔC = Ctct1 – CtGAPDH. A~C are shown for anabolic genes in the AF, catabolic genes as shown in the D~F. (⊗) indicates a significant difference from the internal controls (*p* ≤ 0.05) and (*)indicates a significant difference (*p* ≤ 0.05), and (+) indicates a significant difference (*p* ≤ 0.001)

In-situ immobilization affected the anabolic and catabolic gene expression of discs. The disc gene expression of Group A was consistent with those of the internal control discs. The trend of gene expression including downregulation of collagen II and aggrecan (*p* < 0.05 for both), upregulation of collagen I, MMP3, MMP13, ADAMTs-4 of nucleus pulposus in Group B, C, D, and E, with the exception of group A (*p* < 0.05 all) (Fig. 5A(A-F)). Moreover, downregulation of collagen I (*p* < 0.05), upregulation of collagen II of AF in Group B, C, D, and E, and with the exception of group A(all *p* < 0.05), but did not show a significant effect on gene expression levels of aggrecan, MMP3, MMP13, and ADAMTs-4 of AF in Group A, B, C, D, and E (*p* > 0.05) (Fig. 5B(A-F)).

## Discussion

Abnormal mechanically conditions are crucial contributing factors in IVD degeneration, however genetic factors may also play a significant role [22, 23]. This study is the first to describe the changes in intervertebral discs in different periods of time during in-situ immobilization of caudal vertebrae in rats, based on a macro, micro, and nanoscale change analysis.

The complications of spine immobilization that are associated with halo-vest or a brace treatment have been intensively investigated [24–27]. These studies showed that complications included osteomyelitis and heterotopic ossification etc. It has not yet been analyzed how halo immobilization leads to the complications associated with intervertebral disc degeneration. In our study, we have assessed the effects of long-segment in-situ immobilization on the intervertebral disc of the caudal vertebra, thereby simulating human cervical spine immobilization. Elliott et al. [28] demonstrated a link between intervertebral discs and body weight that was similar in rats and humans. The rat-tail model was chosen as a model [12, 29–31], in our study, the mechanical environment of the IVD that allows for precise control. Thereby we can rule out the effect of mechanical manipulation from other contributing factors.

A retrospective study in which the stress and activity of IVD degeneration was evaluated, demonstrated that mechanical intervention not only plays a role in changing the pressure or activity, however the application device also reduced the activity of the disc, which can result in IVD degeneration [21]. Our study showed that the the disc height and the MRI T2 signal strength of the experimental disc gradually decreased with fixation time.

Previous studies have shown that fixation triggered a degradation pattern in the IVD, such as downregulation of anabolic gene expression [13], and a decrease in glycosaminoglycan content [32]. Our findings further indicated that the effects of immobilization were more significant in the NP compared to the AF. The greatest effect was observed in group E, and combined with the loss of disc height, MRI T2 signal attenuation and IVD elastic modulus gradual decline over time, and confirmed that degeneration of IVD was not only present at the macroscale and microscale, but also at the nanoscale. These data suggested that immobilization can cause early stage degeneration of the IVD that progressed in severity over time. The long-term nature of this study that hypomobility has an effect on gene expression of the NP was more prominent compared to changes found in the AF. This discrepancy may be due to the different periods of immobilization time and abnormal mechanical conditions. In our study, we were the first to show direct evidence of metabolic response in the IVD in vivo associated with long-segment in-situ immobilization.

The AF with a highly organized structure, runs at angles of approximately 60° to the spinal column [33]. Therefore, elastic fibers perform a very crucial role in the total mechanical properties of the AF [34]. In our previous study, we demonstrated the structure and biomechanics at the nanoscale level from different regions of the AF in loaded IVD [20]. This study revealed that the elastic modulus of nucleus pulposus collagen fibrils in group E was markedly decreased compared to group A (control group). Moreover, the elastic modulus was remarkably increased within the AF, and a significant difference was found in the elastic modulus of collagen fibrils over time. The results are consistent with the morphologic changes of NP and AF at the microscale as indicated by hematoxylin/eosin staining. Moreover, the tendency of gene expression of NP and AF were consistent with the former. Therefore, the results indicate that degradation was not only associated with the disorganization at the microscale, but also suggested modification of collagen fibrils at the nanoscale, which would directly change the mechanical environment around the cells of the AF and NP.

From what has been discussed above, in-situ immobilization creates a unique mechanical state that can cause disc degeneration. Previous studies have confirmed that too much or too little pressure/stretch contributes to a catabolism effect on intervertebral discs, in which hypomobility or excessive activities will increase the damage rate, leading to intervertebral disc on the load response of a U-shaped distribution [35]. It is generally accepted that immobilization osteoporosis (IOP) caused by partial fixation after fractures of the lower limb is a common complication in clinical therapeutics. Bed rest and immobilization time are independent predictors of low bone density in the hip [36]. According to Wolff’s law, disuse osteoporosis is thought to be associated with a lack of mechanical forces [37, 38]. We believe that hypomobility or immobilization caused degeneration of muscular ligaments and facet joints in surrounding spine tissue. However, we cannot exclude the possibility that the immobilization apparatus was a factor in the degeneration. Setton et al. showed that hypomobility produced a reduced stimulus to the metabolic activity of disc cells [39]. In an unloaded condition, the IVD swells and starts to lose proteoglycans [40]. Dynamic loading of a certain magnitude, frequency, and duration has been shown to maintain the ECM balance within the disc [41–44]. Static loading induces cell death and causes disc degradation [21, 45–47]. Choi et al. [48] insisted that spine fixation and endplate injury or fracture by internal transpedicular fixation without fusion plays a crucial role in IVD degradation. These studies suggested that hypomobility or immobilization and static loads are both not an innocuous mechanical environment to IVD, in contrast, an adverse effect was found. Ragab et al. [49] showed that cervical fusion resulted in increased strains at adjacent levels. However, long-segment cervical fusions (2 and 3-level fusions) compared to short-segment fusions (1-level fusions), increased the strain approximately 2- to 3-fold compared to the standard. These findings suggested that long-segment fusions or immobilization result in increased side effects compared to short-segment fusions or fixation. This indicates that the long-segment in-situ immobilization triggered a degradation pattern in the IVD. Therefore, in the clinic, it should be possible to reduce spine (cervical spine) long segmental immobilization, and spine (cervical spine) immobilization time (≤6 weeks).

However, a limitation of this study was the difference between a rat tail disc and a human cervical disc in terms of biochemical composition, molecular composition, and biomechanical capabilities. We used the rat caudal vertebral model to simulate human cervical spine IVD changes. While not completely equal to human intervertebral disc changes, the rat model is the currently accepted model and was easier to build and replicate than alternatives. More importantly, it provided relevant experimental specimens and related parameters of IVD that human cervical spine cannot provide. It also provides us with detailed experimental data and theoretical basis for further elaborating the effect of immobilisation on intervertebral disc.

## Conclusions

In this study, long-segment in-situ immobilization caused target disc degeneration, and positively correlated with fixation time. These findings showed that, over time, in-situ immobilization induced a progressive decrease in the intervertebral disc space height and T2 signal intensity of IVD. In addition, downregulation of collagen II, and upregulation of collagen I, aggrecanase, collagenase, stromelysin of NP, and downregulation of collagen I, and upregulation of collagen II of AF were found. However, the effects on aggrecanase, collagenase, stromelysin of AF were not significant. These changes suggested that immobilization loading initiated a degenerative cascade, although this trend was significantly observed in the NP compared to AF. These results as well as the variation tendency of the elastic modulus of collagen fibrils within the NP and AF were confirmed. The discrepancy found with other studies, may be due to the different periods of immobilization and loading conditions. Histological analysis, including hematoxylin/eosin staining indicated that the NP and AF showed a progressive degeneration by in-situ immobilization. However, the comparison with overload conditions was moderate. This study evaluated how hypomobility of the spine may result in IVD degradation and spine lesions. The loading parameters chosen for our study were intended to imitate cervical spine long-segment in-situ immobilization in humans. The results indicated that the degeneration was not only associated with changes at the macroscale, microscale, but also indicated changes in collagen fibrils at the nanoscale, which would directly change the mechanical environment around the cells of the NP and AF. In conclusion, increasing our understanding of the pathogenesis and complications found after long-segment immobilization of the cervical spine, and how to optimize the use of external fixator devices are clearly warranted.

## Abbreviations

AF: Annulus fibrosus
AFM: Atomic Force Microscope
Co: Coccygeal spine
ECM: Extracellular Matrix
EDTA: Ethylenediaminetetraacetic acid
IVD: Intervertebral disc
LBP: Low back pain
NP: Nucleus pulposus
PCR: Polymerase chain reaction
